# Type 2 diabetes mellitus, glycaemic control, associated therapies and risk of rheumatoid arthritis: a retrospective cohort study

**DOI:** 10.1093/rheumatology/keab148

**Published:** 2021-02-16

**Authors:** Dawit T Zemedikun, Krishna Gokhale, Joht Singh Chandan, Jennifer Cooper, Janet M Lord, Andrew Filer, Marie Falahee, Krishnarajah Nirantharakumar, Karim Raza

**Affiliations:** 1 Institute of Applied Health Research, University of Birmingham, Birmingham; 2 Warwick Medical School, University of Warwick, Coventry; 3 Institute of Inflammation and Ageing; 4 MRC Versus Arthritis Centre for Musculoskeletal Ageing Research, University of Birmingham; 5 Sandwell and West Birmingham NHS Hospitals Trust, Birmingham, UK

**Keywords:** type 2 diabetes, rheumatoid arthritis, epidemiology, electronic health records

## Abstract

**Objective:**

To compare the incident risk of RA in patients with type 2 diabetes mellitus (T2DM) and to explore the role of glycaemic control and associated therapeutic use in the onset of RA.

**Methods:**

This study was a retrospective cohort study using patients derived from the IQVIA Medical Research Data (IMRD-UK) database between 1995 and 2019. A total of 224 551 newly diagnosed patients with T2DM were matched to 449 101 patients without T2DM and followed up to assess their risk of RA. Further analyses investigated the effect of glycaemic control, statin use and anti-diabetic drugs on the relationship between T2DM and RA using a time-dependent Cox regression model.

**Results:**

During the study period, the incidence of RA was 8.1 and 10.6 per 10 000 person-years in the exposed and unexposed groups, respectively. The adjusted hazard ratio (aHR) was 0.73 (95% CI 0.67, 0.79). In patients who had not used statins in their lifetime, the aHR was 0.89 (95% CI 0.69, 1.14). When quantifying the effects of glycaemic control, anti-diabetic drugs and statins using time-varying analyses, there was no association with glycaemic control [aHR 1.00 (95% CI 0.99, 1.00)], use of metformin [aHR 1.00 (95% CI 0.82, 1.22)], dipeptidyl peptidase-4 inhibitors [DPP4is; aHR 0.94 (95% CI 0.71, 1.24)] and the development of RA. However, statins demonstrated a protective effect for progression of RA in those with T2DM [aHR 0.76 (95% CI 0.66, 0.88)], with evidence of a duration–response relationship.

**Conclusion:**

There is a reduced risk of RA in patients with T2DM that may be attributable to the use of statins.

**Rheumatology key messages**

- There is a reduced risk of RA in patients with type 2 diabetes compared with non-diabetic controls.
- The use of metformin and/or DPP4i did not influence the risk of RA in patients with type 2 diabetes.
- Statin use in patients with type 2 diabetes was associated with a significantly reduced risk of RA.

## Introduction

RA is a common chronic inflammatory disease of multifactorial aetiology associated with systemic inflammation [1]. RA affects >400 000 people in the UK [2], with an annual incidence estimated at 3.8 cases per 10 000 population [3]. Common symptoms include joint pain, swelling and stiffness, often accompanied by fatigue. Persistent inflammation leads to joint erosion and a loss of function. Extra-articular features, including cardiometabolic and pulmonary disease, are common, even at an early stage [4]. A third of people with RA stop working within 2 years and half are unable to work within 10 years [5].

There is currently no cure for RA, and long-term treatment with DMARDs is usually required to limit disease progression [6]. The potential toxicity and high cost associated with many of these treatments has led to considerable effort to understand the causes of RA and the pathology of its earliest phases with a view to developing preventive interventions [7].

A range of modifiable environmental risk factors are associated with RA [8], with cigarette smoking having the strongest evidence base [9, 10]. A wide range of genetic factors are also known to contribute to the risk of RA [11]. Shared genetic risk factors between RA and other autoimmune diseases such as type 1 diabetes [12–14] explain the documented association between these conditions [15–17]. Current literature suggests that patients with RA have an increased risk of type 2 diabetes mellitus (T2DM) [18], although this association was not evident when controlled for underlying BMI [19]. Interestingly, this relationship may be influenced by medications used in patients with RA [20]. Drugs prescribed in RA modulate diabetes risk with HCQ [21], abatacept [21] and anti-TNF therapy [22] reducing the risk of DM, while glucocorticoids are associated with an increased risk [21].

In contrast, there is limited evidence for an increased risk of RA following a diagnosis of T2DM. One may hypothesize that RA would be more common in patients with T2DM due to shared risk factors, including obesity, metabolic syndrome and chronic low-grade inflammation [23, 24]. A cross-sectional study from Taiwan found a 46% higher risk of developing RA in women but not in men with a diagnosis of T2DM [25]. The European Prospective Investigation of Cancer-Norfolk and the Norfolk Arthritis Register (EPIC-2-NOAR), a longitudinal community-based study, reported a 2.5-fold increased risk of inflammatory polyarthritis in patients with self-reported diabetes, but type 1 and 2 diabetes were not differentiated. Furthermore, only 11 patients who developed inflammatory polyarthritis had diabetes at baseline [26].

Many drugs used to treat T2DM have immunomodulatory properties, and those need to be taken into account when exploring the risk of RA in patients with T2DM. For example, dipeptidyl peptidase-4 inhibitors (DDP4is) achieve their anti-hyperglycaemic effect through prevention of DDP4-mediated degradation of incretin hormones [including glucagon-like peptide-1 (GLP-1)]. In addition to their anti-hyperglycaemic effects, DPP4is also have anti-inflammatory properties [27]. Two studies using insurance claims–based data suggested a 33% reduced risk of RA in patients treated with DPP4is, although those studies failed to adjust for important confounders, including BMI [28, 29]. A subsequent time-dependent analysis using the Clinical Practice Research Datalink (CPRD), which adjusted for known risk factors for RA including BMI and smoking status, demonstrated no evidence of association between DPP4is and the incidence of RA [hazard ratio (HR) 1.0 (95% CI 0.8, 1.3)] [30]. The use of metformin, which also has anti-inflammatory properties [31], has been associated with a reduced risk of RA [32]. Previous studies assessing the effects of statins on RA development have reported conflicting results, with one study reporting an increased risk [33] and others reporting a reduced risk [34, 35]. A recent systematic review identified no difference in RA risk between statin users and non-users, although methodological limitations of the included studies were identified [36]. However, the effect of statins on RA risk in patients with T2DM remains unknown.

We therefore aimed to provide a definitive answer to the question of whether T2DM is associated with an increased onset of RA and to explore the role of glycaemic control in the risk of RA and whether anti-diabetic drugs (oral and injectable) and lipid-lowering drugs (specifically statins) influence the risk of RA.

## Methods

### Study design and data source

This study was a population-based retrospective open cohort study using patient data derived from the IQVIA Medical Research Data (IMRD-UK), formerly the Health Improvement Network (THIN) database. The IMRD-UK is a nationally representative electronic primary care database that contains pseudo-anonymized medical records for >15 million patients derived from 808 general practices in the UK. The IMRD-UK has been demonstrated to be representative of the UK population in terms of demographic structure and common morbidity prevalence [37]. The database has been used in numerous epidemiological studies to examine health outcomes in T2DM [38–40] and RA [41, 42]. Information relating to symptoms, examinations, investigations and diagnoses are recorded within the IMRD-UK as Read codes, a clinical hierarchy coding system [43]. To reduce underrecording of events and improve data quality, general practices were included 12 months after they installed electronic medical records or from the practice’s acceptable mortality recording (AMR) date [44–46].

### Study population, exposure and outcome

The study period was set between 1 January 1995 and 31 December 2019. Adult patients ≥18 years of age registered for at least 12 months with any of the eligible practices formed the source population. The exposed cohort consisted of incident cases (newly diagnosed patients) with T2DM. Exposure to T2DM was ascertained by the presence of Read codes indicative of diagnosis (Supplementary Table S1, available at *Rheumatology* online) in the patient’s medical record and the absence of any type 1 diabetes diagnostic code. The outcome, RA, was also defined on the basis of Read codes (Supplementary Table S1, available at *Rheumatology* online). Codes relating to diabetes and the outcome of RA are part of the Quality Outcomes Framework (QOF), a payment incentivized coding system for general practitioners (GPs) within the UK [47]. These diagnoses have been validated in primary care settings [48]. Each exposed patient was matched to up to two unexposed control patients who were randomly selected from an age- and sex-matched pool of eligible patients without a record of T2DM at any time.

### Follow-up period

The index date for the exposed patients was the date of the first recorded Read code relating to T2DM exposure once a patient was eligible to take part in the study. To avoid immortal time bias [49], the same index date was assigned to the corresponding unexposed patient. Both exposed and unexposed patients were followed up from the index date until the earliest of the following endpoints that defined the exit date: outcome (RA) date, study end date, last date of data collection from a given GP, date patient transferred from GP and death date.

A 15 month latency period (lag period ensuring the index date was set 15 months following the date of diagnosis) was included in the selection of exposed patients. This was to ensure that all covariates predicting the risk of RA in patients with T2DM were recorded at baseline as per QOF guidelines [50, 51]. The latency period also limited the possibility of silent RA preceding T2DM being misclassified as incident RA, reducing the likelihood of reverse causality in our study.

### Effect on RA risk of glycaemic control and medication in patients with DM

In order to account for the differential impact of medications (defined through drug codes) acting as modifiers along the pathway, three variations of the study design were used to examine the relationship between T2DM and the risk of developing RA.

The first study included all eligible exposed patients who were matched by age (±1 year) and sex to up to two unexposed patients. The aim of this study was to look at the overall risk of RA in newly diagnosed patients with T2DM.

The second study aimed at assessing the impact of statin use on the onset of RA by replicating the first study, but in patients who at no time point in the database had a prescription for statins (both in exposed and unexposed patients).

A time-dependent approach was adopted in the third study by taking only patients with prescribed anti-diabetic medication from the first study. In this study, patients entered the cohort on receiving a prescription of an anti-diabetic drug. Longitudinal measurements during the study period were collected for these patients to assess the effect of glycaemic control and differential effects of anti-diabetic drugs (particularly metformin) and statins on subsequent RA risk.

### Covariates

Known confounders and relevant covariates based on biological plausibility were used in the adjusted analyses. These included age, sex, BMI, smoking status, ethnicity and deprivation assessed by the Townsend deprivation quintiles. The Townsend score is calculated using social indices such as income, education and employment. All baseline data used were the latest recorded on the index date and all subsequent records until the patients exited the study (for time-varying covariates). In the third variation of the study, glycaemic control, statins and anti-diabetic drugs were treated as time-varying covariates measured in 3 month intervals.

### Statistical analysis

Baseline characteristics of the cohorts were reported using appropriate descriptive statistics. In order to calculate an incidence rate (IR) per 10 000 person-years (py) for each of the outcomes of interest, patients with pre-existing RA were excluded when extracting data to ensure the IR reflected outcomes that occurred following cohort entry. Cox regression was used in the static models (studies 1 and 2: matched overall cohort and cohort devoid of statin use) to calculate crude and adjusted HRs (aHRs) together with their corresponding 95% CIs comparing the incidence of RA in patients with and without T2DM. Subgroup analyses were conducted to assess sex-specific differences.

We used extended Cox proportional hazards models in the time-dependent analyses (study 3). Time-varying covariance occurs when a given covariate changes as a function of time during the follow-up period [52]. The main approach for survival analysis with a time-varying covariate is time-dependent Cox regression modelling, which extends the Cox proportional hazard model to allow time-varying covariates [53]. For this, it was essential to organize the data in a counting process style with a fixed follow-up interval (3 months in this case) for each individual. In the primary model we assessed the effect of glycaemic control [haemoglobin A1c (HbA1c)], metformin against any other oral hypoglycaemic agent and statin use against the absence of a prescription for a statin. Thereafter, to validate our finding, we used the same method but with DPP4i as the exposure of interest instead of metformin. DPP4i has been previously shown to have no effect on the risk of RA in a time-dependent analysis from a similar database [30].

We conducted a sensitivity analysis in the time-dependent analysis for metformin by adding a 3 month lag (one interval) period for drug exposures. This meant that outcomes were considered in the following 3 month interval for the current exposure period to allow sufficient time for the drugs to have an effect. A further sensitivity analysis was performed to assess duration–response relations based on the cumulative duration of statin use. This categorical time-varying variable was defined as the time between the first-ever statin prescription and the time of the event. We used R version 3.4.1 (R Foundation for Statistical Computing, Vienna, Austria) for the time-dependent analyses; all other analyses were performed using Stata SE 16.1 (StataCorp, College Station, TX, USA). The study protocol was approved by the Scientific Review Committee of the data provider, IQVIA (reference number 20SRC016).

## Results

### Baseline characteristics

We identified a total of 224 551 exposed patients (incident T2DM) who were matched to 449 101 unexposed patients in the main cohort (study 1: patients with T2DM compared with patients without T2DM). The mean age at the index date of the cohort was 63 years and 56% were male. During the study period, the median follow-up periods were 4.51 years [interquartile range (IQR) 2.01–7.97] and 3.44 years (IQR 1.43–6.61) for the exposed and unexposed groups, respectively. There was a higher proportion of patients who were obese (BMI >30 kg/m^2^) and in a more deprived socio-economic group in the exposed group than the unexposed group. Baseline characteristics are described in more detail in Table 1.

**Table 1:** Baseline characteristics of the study population

Characteristics	Exposed (*n* = 224 551)	Unexposed (*n* = 449 101)
Sex, *n* (%)		
Male	125 558 (55.92)	251 116 (55.92)
Female	98 993 (44.08)	197 985 (44.08)
Age, years, mean (s.d.)	63.10 (13.16)	63.08 (13.17)
Age categories (years), *n* (%)		
18–34	3786 (1.69)	7651 (1.70)
35–44	15 352 (6.84)	30 847 (6.87)
45–54	39 947 (17.79)	80 020 (17.82)
55–64	59 087 (26.31)	118 253 (26.33)
65–74	59 844 (26.65)	119 284 (26.56)
≥75	46 535 (20.72)	93 046 (20.72)
BMI, kg/m^2^, median (IQR)	30.00 (26.00–34.00)	26.00 (23.00–29.00)
BMI categories (kg/m^2^), *n* (%)		
Underweight (<18.5)/normal weight (18.5–24.9)	30 340 (13.51)	144 989 (32.28)
Overweight (25–29.9)	72 597 (32.33)	149 227 (33.23)
Obese (≥30)	116 343 (51.81)	83 335 (18.56)
Missing or implausible	5271 (2.35)	71 550 (15.93)
Townsend quintiles, *n* (%)		
1 (least deprived)	41 356 (18.42)	102 212 (22.76)
2	39 613 (17.64)	88 768 (19.77)
3	41 212 (18.35)	79 011 (17.59)
4	38 484 (17.14)	63 307 (14.10)
5 (most deprived)	29 271 (13.04)	42 484 (9.46)
Missing	34 615 (15.42)	73 319 (16.33)
Smoking status, *n* (%)		
Non-smoker	105 592 (47.02)	229 049 (51.00)
Smoker	37 007 (16.48)	78 505 (17.48)
Ex-smoker	80 244 (35.74)	117 246 (26.11)
Missing	1708 (0.76)	24 301 (5.41)
Ethnicity, *n* (%)		
White	97 739 (43.53)	186 869 (41.61)
Mixed race	1456 (0.65)	2278 (0.51)
Other	515 (0.23)	852 (0.19)
Black	2987 (1.33)	3442 (0.77)
South Asian	6922 (3.08)	4913 (1.09)
Missing	114 932 (51.18)	250 747 (55.83)

When examining patients without any history of statin use (study 2), there were 40 879 eligible exposed patients who were matched to 81 757 unexposed patients. The mean age was 61 years and 53% were male. The proportions of other covariates were similar to the main cohort and are also described in Supplementary Table S2, available at *Rheumatology* online. The study population in the time-dependent analysis (study 3) included 191 862 patients who had been prescribed anti-diabetic medication in their record. The mean age was 61 years, 57% were male and the median follow-up of the cohort was 4.8 years.

### Risk of incident RA

During the study period, there were 971 (IR 8.1/10 000 py) new diagnoses of RA in the exposed group compared with 2117 (IR 10.6/10 000 py) in the unexposed group (Table 2). Following adjustment, this translated into an HR of 0.73 (95% CI 0.67, 0.79). The reduced risk of incident RA in patients with T2DM compared with controls remained significant when male and female patients were analysed separately (Supplementary Table S3, available at *Rheumatology* online). Once we excluded all patients in the analysis who had a history of statin use, the aHR increased to 0.89 (95% CI 0.69, 1.14) and no significant difference was seen between the groups (Supplementary Table S4, available at *Rheumatology* online).

**Table 2:** Crude and adjusted HRs for the risk of RA in patients with diabetes compared with those without diabetes

Characteristics	Exposed (*n* = 224 551)	Unexposed (*n* = 449 101)
Outcome events, *n* (%)	971 (0.43)	2117 (0.47)
Person-years	1 200 042	1 999 301
Crude incidence rate/10 000 py	8.1	10.6
Follow-up years, median (IQR)	4.51 (2.01–7.97)	3.44 (1.43–6.61)
Unadjusted HR (95% CI)	0.78 (0.72, 0.84)	
*P*-value	<0.01	
Adjusted HR (95% CI)	0.73 (0.67, 0.79)	
*P*-value	<0.01	

Model was adjusted for sex, age, BMI, Townsend deprivation quintiles, smoking status and ethnicity.

### Effects of glycaemic control, anti-diabetic drugs and statins on RA risk

**Fig. 1:**
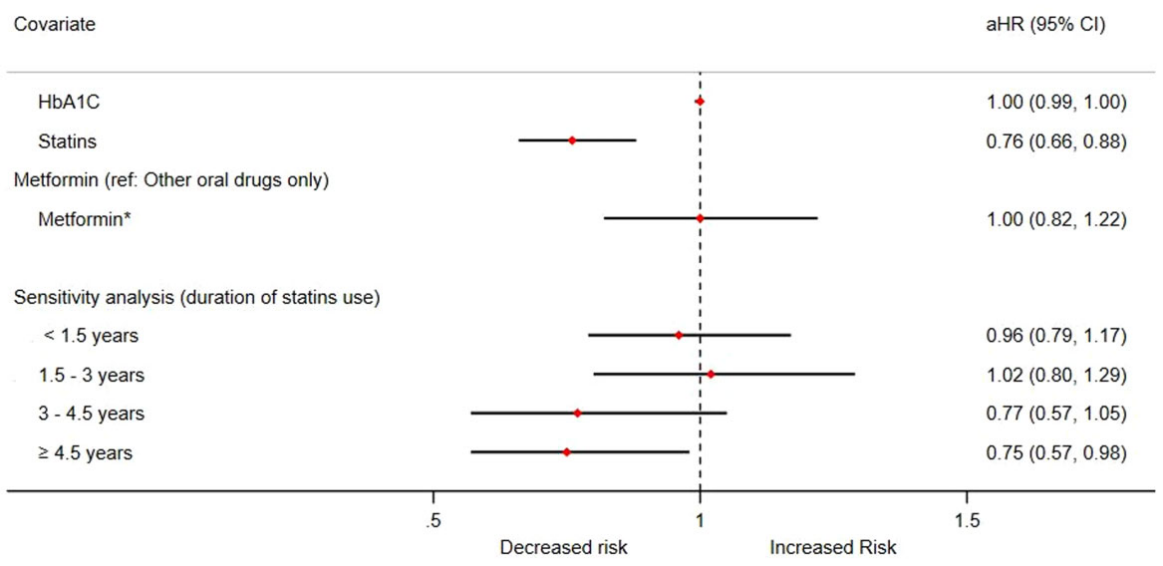
Forest plot summarizing aHRs of glycaemic control and associated therapies on the risk of RA using time-dependent analysis. *Metformin with or without other oral drugs. Models were adjusted for sex, age, BMI, estimated glomerular filtration rate (eGFR), systolic blood pressure, Townsend deprivation quintiles, smoking status, ethnicity, peripheral neuropathy, retinopathy, diabetic foot ulcer, hypothyroidism and cardiovascular disease (CVD).

In study 3, we used time-varying covariates to quantify the effects of glycaemic control, anti-diabetic drugs and statins on the incidence of RA. Fig. 1 presents the key results of the main analysis and the sensitivity analysis. There was no evidence of an association between glycaemic control and the development of RA [aHR 1.00 (95% CI 0.99, 1.00)] in patients with T2DM. Compared with other oral drugs only, the use of metformin with or without other oral drugs was not associated with an altered risk of RA [aHR 1.00 (95% CI 0.82, 1.22)]. On the other hand, the use of statins appeared to significantly reduce the risk of RA in patients with diabetes [aHR 0.76 (95% CI 0.66, 0.88)]. Female patients had a significantly higher risk of developing RA [aHR 1.70 (95% CI 1.48, 1.96)], while the effects of unit increase in age [aHR 1.01 (95% CI 1.00, 1.02)] and BMI [aHR 1.01 (95% CI 1.00, 1.02)] were minimal. We also observed a higher risk with smoking, higher comorbidity score and hypothyroidism. Further details on the results are noted in Supplementary Table S5, available at *Rheumatology* online.

In a sensitivity analysis introducing a duration–response analysis for statins use, the aHRs for risk of RA were evident only after 3 years worth of prescriptions [3–4.5 years: aHR 0.77 (95% CI 0.57, 1.05)] and ≥4.5 years [aHR 0.75 (95% CI 0.57, 0.98)] (Fig. 1 and Supplementary Table S6, available at *Rheumatology* online).

Validation analysis comparing a previous study [30] on the risk of RA in those prescribed DPP4i with and earlier study obtained similar results [aHR 0.94 (95% CI 0.71,1.24)] (Supplementary Table S7, available at *Rheumatology* online).

## Discussion

### Summary of key findings

To our knowledge, this is the first study to investigate the risk of incident RA in patients with T2DM using a UK population-based cohort. Analysis of this large set of electronic health records suggested that patients with T2DM had a reduced risk of developing RA compared with patients without T2DM. However, subgroup analyses limited to patients who were not on statins identified that this decreased risk of incident RA was largely explained by the use of statins. Our study is the first to examine the effects of longitudinally measured HbA1c, metformin and statins on the incidence of RA using a time-varying covariate approach. Neither glycaemic control nor any of the anti-diabetic medications used, including metformin and DPP4i, influenced the risk of RA in patients with T2DM. The use of statins reduced the risk of RA.

### In context with current literature

Our findings conflict with those of Lu *et al.* [25], who observed an increased risk of RA in female patients with T2DM, but they did not control for confounding variables such as BMI and medication use. The present study does not support an association between increased risk of RA in patients with T2DM. In fact, we found a lower incidence that was potentially attributable to the use of statins in patients with T2DM.

Emerging evidence supports the hypothesis that metformin interferes with key immunopathological mechanisms in systemic autoimmune diseases [54]. However, in our study there was absence of an association between metformin use and RA risk. Our finding differs from the results of the study by Naffaa *et al.* [32], who found that adherence to metformin treatment was associated with a decreased risk of RA in female but not male patients. That study measured adherence by calculating the duration of metformin prescription and reporting it as a proportion of the follow-up duration. Furthermore, they did not find a dose–response relationship. Indeed, compared with the lowest adherence group (<20%), the highest adherence group (>80%) had the lowest effect size. However, their study did not fully adjust for BMI, a common risk factor for both T2DM and pro-inflammatory disorders. Adjustment was made for the presence of obesity (BMI >30 kg/m^2^), and obesity was associated with a reduced risk of RA, which contrasts with our findings and those of a recent meta-analysis [55].

Douros *et al.* [30] used a time-varying exposure definition to model the impact of DPP4i on the onset of RA in patients with T2DM using data from another UK primary care database (CPRD). In line with our findings, they showed that the use of DPP4i, compared with other anti-diabetic drugs, was not associated with an altered risk of incident RA [HR 1.0 (95% CI 0.8, 1.3)]. Using US health claims data, one previous study reported a decreased risk of incident RA in DPP4i initiators [aHR 0.66 (95% CI 0.44, 0.99)] when compared with the use of second-line oral anti-diabetic drugs [28]. However, that study had a short duration of follow-up of ∼9 months and did not account for statin use, which our study suggests is responsible for the apparent decreased risk of RA in patients with T2DM.

A previous systematic review [36] found no difference in the risk of RA in statin users *vs* non-users, but a lower risk of RA was associated with the use of higher doses of statins or greater persistence with statin treatment. The findings of our study extend this by showing a protective effect of statin use against the progression of RA in those with T2DM and are consistent with evidence for an anti-inflammatory effect of statins. RA is characterized by a progressive chronic inflammatory response associated with high levels of pro-inflammatory cytokines, including TNF-α, IL-1 and IL-6 [1, 6]. Indeed, TNF-α inhibitors are a key group of DMARDs used to limit disease progression in RA [6]. Statin use in patients with established RA has been shown both clinically and experimentally to reduce serum levels of pro-inflammatory cytokines as well as CRP [56–58]. T2DM is also strongly associated with elevated levels of IL-6, CRP and TNF-α, an effect that remains statistically significant even when controlling for BMI [59]. There is also a linear association between glycaemic control and IL-6 levels [59]. It is possible that attenuation of these inflammatory pathways by statins drives the effect we found for patients with T2DM. However, further exploration of these mechanisms is needed [58]. Thus our findings provide justification for a trial to assess whether statins can prevent the development of RA in individuals at high risk of RA and for an increased understanding of the preferences of those at risk of RA for preventive treatment [60].

### Strengths and limitations

This study used large sample sizes from the IMRD-UK database, which is generalizable to the UK population. Electronic health records data may be subject to potential biases resulting from misdiagnoses and inconsistent or incomplete coding of medical conditions. Nevertheless, both RA and diabetes are in the QOF domain, where recording quality is generally high. The prevalence of major chronic conditions in IMRD-UK, adjusted for patient demographics, are also similar to national estimates [37]. On the other hand, there may be residual confounding and therefore the findings should be interpreted cautiously.

## Conclusion

RA is a common chronic disease with high personal, societal and healthcare costs. While good disease control can now be achieved in many patients, the inconvenience, risk and expense associated with long-term therapies is considerable. Over the last 10 years there has been an increased focus on understanding risk factors for RA with a view to developing and testing preventive interventions [61, 62]. Understanding the impact on RA onset of other morbid conditions and of commonly used drugs that may have an impact on immune function are thus important. This study found reduced risk of RA in patients with T2DM, but this did not persist when statin users were excluded. The use of metformin and DPP4i did not influence the risk of RA. However, the results suggest that statin use may be associated with a reduced risk of RA, a finding that needs further investigation in a trial setting.

## Supplementary Material

keab148_Supplementary_Data
