# Supplementary material for: Type 2 diabetes mellitus, glycaemic control, associated therapies and risk of rheumatoid arthritis: a retrospective cohort study
Source: Rheumatology (Oxford). 2021 Feb 16;60(12):5567–75. doi: 10.1093/rheumatology/keab148 (PMC8645277; doi:10.1093/rheumatology/keab148)
Supplement: keab148_Supplementary_Data [file keab148_supplementary_data.docx]

# Supplementary material

**Supplementary Table S1: Read codes for exposure and outcome definitions**

**Type 2 diabetes**

| **Clinical code** | **Description** |
| --- | --- |
| C10..00 | Diabetes mellitus |
| C100.00 | Diabetes mellitus with no mention of complication |
| C100100 | Diabetes mellitus, adult onset, no mention of complication |
| C100112 | Non-insulin dependent diabetes mellitus |
| C100z00 | Diabetes mellitus NOS with no mention of complication |
| C101100 | Diabetes mellitus, adult onset, with ketoacidosis |
| C101y00 | Other specified diabetes mellitus with ketoacidosis |
| C102.00 | Diabetes mellitus with hyperosmolar coma |
| C102100 | Diabetes mellitus, adult onset, with hyperosmolar coma |
| C102z00 | Diabetes mellitus NOS with hyperosmolar coma |
| C103y00 | Other specified diabetes mellitus with coma |
| C104.00 | Diabetes mellitus with renal manifestation |
| C104.11 | Diabetic nephropathy |
| C104100 | Diabetes mellitus, adult onset, with renal manifestation |
| C104y00 | Other specified diabetes mellitus with renal complications |
| C104z00 | Diabetes mellitus with nephropathy NOS |
| C105.00 | Diabetes mellitus with ophthalmic manifestation |
| C105100 | Diabetes mellitus, adult onset, + ophthalmic manifestation |
| C105y00 | Other specified diabetes mellitus with ophthalmic complication |
| C105z00 | Diabetes mellitus NOS with ophthalmic manifestation |
| C106.00 | Diabetes mellitus with neurological manifestation |
| C106.11 | Diabetic amyotrophy |
| C106.12 | Diabetes mellitus with neuropathy |
| C106.13 | Diabetes mellitus with polyneuropathy |
| C106100 | Diabetes mellitus, adult onset, + neurological manifestation |
| C106y00 | Other specified diabetes mellitus with neurological comps |
| C106z00 | Diabetes mellitus NOS with neurological manifestation |
| C107.00 | Diabetes mellitus with peripheral circulatory disorder |
| C107.11 | Diabetes mellitus with gangrene |
| C107.12 | Diabetes with gangrene |
| C107100 | Diabetes mellitus, adult, + peripheral circulatory disorder |
| C107200 | Diabetes mellitus, adult with gangrene |
| C107400 | NIDDM with peripheral circulatory disorder |
| C107y00 | Other specified diabetes mellitus with periph circ comps |
| C107z00 | Diabetes mellitus NOS with peripheral circulatory disorder |
| C108y00 | Other specified diabetes mellitus with multiple comps |
| C108z00 | Unspecified diabetes mellitus with multiple complications |
| C109.00 | Non-insulin dependent diabetes mellitus |
| C109.11 | NIDDM - Non-insulin dependent diabetes mellitus |
| C109.12 | Type 2 diabetes mellitus |
| C109.13 | Type II diabetes mellitus |
| C109000 | Non-insulin-dependent diabetes mellitus with renal comps |
| C109011 | Type II diabetes mellitus with renal complications |
| C109012 | Type 2 diabetes mellitus with renal complications |
| C109100 | Non-insulin-dependent diabetes mellitus with ophthalm comps |
| C109111 | Type II diabetes mellitus with ophthalmic complications |
| C109112 | Type 2 diabetes mellitus with ophthalmic complications |
| C109200 | Non-insulin-dependent diabetes mellitus with neuro comps |
| C109211 | Type II diabetes mellitus with neurological complications |
| C109212 | Type 2 diabetes mellitus with neurological complications |
| C109300 | Non-insulin-dependent diabetes mellitus with multiple comps |
| C109311 | Type II diabetes mellitus with multiple complications |
| C109312 | Type 2 diabetes mellitus with multiple complications |
| C109400 | Non-insulin dependent diabetes mellitus with ulcer |
| C109411 | Type II diabetes mellitus with ulcer |
| C109412 | Type 2 diabetes mellitus with ulcer |
| C109500 | Non-insulin dependent diabetes mellitus with gangrene |
| C109511 | Type II diabetes mellitus with gangrene |
| C109512 | Type 2 diabetes mellitus with gangrene |
| C109600 | Non-insulin-dependent diabetes mellitus with retinopathy |
| C109611 | Type II diabetes mellitus with retinopathy |
| C109612 | Type 2 diabetes mellitus with retinopathy |
| C109700 | Non-insulin dependent diabetes mellitus - poor control |
| C109711 | Type II diabetes mellitus - poor control |
| C109712 | Type 2 diabetes mellitus - poor control |
| C109900 | Non-insulin-dependent diabetes mellitus without complication |
| C109912 | Type 2 diabetes mellitus without complication |
| C109A00 | Non-insulin dependent diabetes mellitus with mononeuropathy |
| C109B00 | Non-insulin dependent diabetes mellitus with polyneuropathy |
| C109B11 | Type II diabetes mellitus with polyneuropathy |
| C109B12 | Type 2 diabetes mellitus with polyneuropathy |
| C109C00 | Non-insulin dependent diabetes mellitus with nephropathy |
| C109C11 | Type II diabetes mellitus with nephropathy |
| C109C12 | Type 2 diabetes mellitus with nephropathy |
| C109D00 | Non-insulin dependent diabetes mellitus with hypoglyca coma |
| C109D11 | Type II diabetes mellitus with hypoglycaemic coma |
| C109D12 | Type 2 diabetes mellitus with hypoglycaemic coma |
| C109E00 | Non-insulin depend diabetes mellitus with diabetic cataract |
| C109E11 | Type II diabetes mellitus with diabetic cataract |
| C109E12 | Type 2 diabetes mellitus with diabetic cataract |
| C109F00 | Non-insulin-dependent d m with peripheral angiopath |
| C109F11 | Type II diabetes mellitus with peripheral angiopathy |
| C109F12 | Type 2 diabetes mellitus with peripheral angiopathy |
| C109G00 | Non-insulin dependent diabetes mellitus with arthropathy |
| C109G12 | Type 2 diabetes mellitus with arthropathy |
| C109H00 | Non-insulin dependent d m with neuropathic arthropathy |
| C109H11 | Type II diabetes mellitus with neuropathic arthropathy |
| C109H12 | Type 2 diabetes mellitus with neuropathic arthropathy |
| C109J00 | Insulin treated Type 2 diabetes mellitus |
| C109J11 | Insulin treated non-insulin dependent diabetes mellitus |
| C109J12 | Insulin treated Type II diabetes mellitus |
| C109K00 | Hyperosmolar non-ketotic state in type 2 diabetes mellitus |
| C10A.00 | Malnutrition-related diabetes mellitus |
| C10A500 | Malnutritn-relat diabetes melitus wth periph circul complctn |
| C10B.00 | Diabetes mellitus induced by steroids |
| C10B000 | Steroid induced diabetes mellitus without complication |
| C10C.00 | Diabetes mellitus autosomal dominant |
| C10D.00 | Diabetes mellitus autosomal dominant type 2 |
| C10ER00 | Latent autoimmune diabetes mellitus in adult |
| C10F.00 | Type 2 diabetes mellitus |
| C10F.11 | Type II diabetes mellitus |
| C10F000 | Type 2 diabetes mellitus with renal complications |
| C10F011 | Type II diabetes mellitus with renal complications |
| C10F100 | Type 2 diabetes mellitus with ophthalmic complications |
| C10F111 | Type II diabetes mellitus with ophthalmic complications |
| C10F200 | Type 2 diabetes mellitus with neurological complications |
| C10F211 | Type II diabetes mellitus with neurological complications |
| C10F300 | Type 2 diabetes mellitus with multiple complications |
| C10F311 | Type II diabetes mellitus with multiple complications |
| C10F400 | Type 2 diabetes mellitus with ulcer |
| C10F411 | Type II diabetes mellitus with ulcer |
| C10F500 | Type 2 diabetes mellitus with gangrene |
| C10F511 | Type II diabetes mellitus with gangrene |
| C10F600 | Type 2 diabetes mellitus with retinopathy |
| C10F611 | Type II diabetes mellitus with retinopathy |
| C10F700 | Type 2 diabetes mellitus - poor control |
| C10F711 | Type II diabetes mellitus - poor control |
| C10F900 | Type 2 diabetes mellitus without complication |
| C10F911 | Type II diabetes mellitus without complication |
| C10FA00 | Type 2 diabetes mellitus with mononeuropathy |
| C10FA11 | Type II diabetes mellitus with mononeuropathy |
| C10FB00 | Type 2 diabetes mellitus with polyneuropathy |
| C10FB11 | Type II diabetes mellitus with polyneuropathy |
| C10FC00 | Type 2 diabetes mellitus with nephropathy |
| C10FC11 | Type II diabetes mellitus with nephropathy |
| C10FD00 | Type 2 diabetes mellitus with hypoglycaemic coma |
| C10FD11 | Type II diabetes mellitus with hypoglycaemic coma |
| C10FE00 | Type 2 diabetes mellitus with diabetic cataract |
| C10FE11 | Type II diabetes mellitus with diabetic cataract |
| C10FF00 | Type 2 diabetes mellitus with peripheral angiopathy |
| C10FF11 | Type II diabetes mellitus with peripheral angiopathy |
| C10FG00 | Type 2 diabetes mellitus with arthropathy |
| C10FG11 | Type II diabetes mellitus with arthropathy |
| C10FH00 | Type 2 diabetes mellitus with neuropathic arthropathy |
| C10FH11 | Type II diabetes mellitus with neuropathic arthropathy |
| C10FJ00 | Insulin treated Type 2 diabetes mellitus |
| C10FJ11 | Insulin treated Type II diabetes mellitus |
| C10FK00 | Hyperosmolar non-ketotic state in type 2 diabetes mellitus |
| C10FK11 | Hyperosmolar non-ketotic state in type II diabetes mellitus |
| C10FL00 | Type 2 diabetes mellitus with persistent proteinuria |
| C10FL11 | Type II diabetes mellitus with persistent proteinuria |
| C10FM00 | Type 2 diabetes mellitus with persistent microalbuminuria |
| C10FM11 | Type II diabetes mellitus with persistent microalbuminuria |
| C10FN00 | Type 2 diabetes mellitus with ketoacidosis |
| C10FN11 | Type II diabetes mellitus with ketoacidosis |
| C10FP00 | Type 2 diabetes mellitus with ketoacidotic coma |
| C10FP11 | Type II diabetes mellitus with ketoacidotic coma |
| C10FQ00 | Type 2 diabetes mellitus with exudative maculopathy |
| C10FQ11 | Type II diabetes mellitus with exudative maculopathy |
| C10FR00 | Type 2 diabetes mellitus with gastroparesis |
| C10FR11 | Type II diabetes mellitus with gastroparesis |
| C10FS00 | Maternally inherited diabetes mellitus |
| C10G.00 | Secondary pancreatic diabetes mellitus |
| C10G000 | Secondary pancreatic diabetes mellitus without complication |
| C10H.00 | Diabetes mellitus induced by non-steroid drugs |
| C10H000 | DM induced by non-steroid drugs without complication |
| C10K.00 | Type A insulin resistance |
| C10K000 | Type A insulin resistance without complication |
| C10M.00 | Lipoatrophic diabetes mellitus |
| C10N000 | Secondary diabetes mellitus without complication |
| C10N100 | Cystic fibrosis related diabetes mellitus |
| C10P.00 | Diabetes mellitus in remission |
| C10P100 | Type II diabetes mellitus in remission |
| C10P111 | Type 2 diabetes mellitus in remission |
| C10y.00 | Diabetes mellitus with other specified manifestation |
| C10y100 | Diabetes mellitus, adult, + other specified manifestation |
| C10yy00 | Other specified diabetes mellitus with other spec comps |
| C10yz00 | Diabetes mellitus NOS with other specified manifestation |
| C10z.00 | Diabetes mellitus with unspecified complication |
| C10z100 | Diabetes mellitus, adult onset, + unspecified complication |
| C10zy00 | Other specified diabetes mellitus with unspecified comps |
| C10zz00 | Diabetes mellitus NOS with unspecified complication |

**Rheumatoid arthritis**

| **Clinical code** | **Description** |
| --- | --- |
| N040.00 | Rheumatoid arthritis |
| N040000 | Rheumatoid arthritis of cervical spine |
| N040100 | Other rheumatoid arthritis of spine |
| N040200 | Rheumatoid arthritis of shoulder |
| N040300 | Rheumatoid arthritis of sternoclavicular joint |
| N040400 | Rheumatoid arthritis of acromioclavicular joint |
| N040500 | Rheumatoid arthritis of elbow |
| N040600 | Rheumatoid arthritis of distal radio-ulnar joint |
| N040700 | Rheumatoid arthritis of wrist |
| N040800 | Rheumatoid arthritis of MCP joint |
| N040900 | Rheumatoid arthritis of PIP joint of finger |
| N040A00 | Rheumatoid arthritis of DIP joint of finger |
| N040B00 | Rheumatoid arthritis of hip |
| N040C00 | Rheumatoid arthritis of sacro-iliac joint |
| N040D00 | Rheumatoid arthritis of knee |
| N040E00 | Rheumatoid arthritis of tibio-fibular joint |
| N040F00 | Rheumatoid arthritis of ankle |
| N040G00 | Rheumatoid arthritis of subtalar joint |
| N040H00 | Rheumatoid arthritis of talonavicular joint |
| N040J00 | Rheumatoid arthritis of other tarsal joint |
| N040K00 | Rheumatoid arthritis of 1st MTP joint |
| N040L00 | Rheumatoid arthritis of lesser MTP joint |
| N040M00 | Rheumatoid arthritis of IP joint of toe |
| N040N00 | Rheumatoid vasculitis |
| N040P00 | Seronegative rheumatoid arthritis |
| N040Q00 | Rheumatoid bursitis |
| N040R00 | Rheumatoid nodule |
| N040S00 | Rheumatoid arthritis - multiple joint |
| N040T00 | Flare of rheumatoid arthritis |

**Supplementary Table S2: Baseline characteristics of the no-statins study population**

| **No-statins cohort** | **Exposed** | **Unexposed** |
| --- | --- | --- |
| All | n = 40,879 | n = 81,757 |
| Sex (%) |  |  |
| Male | 21653 (52.97) | 43306 (52.97) |
| Female | 19226 (47.03) | 38451 (47.03) |
| Age in years (mean (sd)) | 60.75 (16.61) | 60.78 (16.61) |
| Age Categories in years (%) |  |  |
| 18-34 | 2176 (5.32) | 4389 (5.37) |
| 35-44 | 5157 (12.62) | 10328 (12.63) |
| 45-54 | 8314 (20.34) | 16613 (20.32) |
| 55-64 | 8251 (20.18) | 16518 (20.20) |
| 65-74 | 7043 (17.23) | 14054 (17.19) |
| 75 & overs | 9938 (24.31) | 19855 (24.29) |
| BMI- kg/m^2^ (mean (sd)) | 31.61 (7.57) | 26.41 (5.08) |
| BMI Categories- kg/m^2^ (%) |  |  |
| Underweight (<18.5)/Normal weight (18.5-24.9) | 6695 (16.38) | 28653 (35.05) |
| Overweight (25-29.9) | 11691 (28.60) | 24393 (29.84) |
| Obese (30 & above) | 20499 (50.15) | 13242 (16.20) |
| Missing or implausible | 1994 (4.88) | 15469 (18.92) |
| Townsend quintiles (%) |  |  |
| 1 (Least deprived) | 7254 (17.75) | 18006 (22.02) |
| 2 | 7063 (17.28) | 15834 (19.37) |
| 3 | 7289 (17.83) | 14463 (17.69) |
| 4 | 6927 (16.95) | 11543 (14.12) |
| 5 (Most deprived) | 4953 (12.12) | 7554 (9.24) |
| Missing | 7393 (18.09) | 14357 (17.56) |
| Smoking Status (%) |  |  |
| Non-smoker | 6065 (14.84) | 13608 (16.64) |
| Smoker | 22311 (54.58) | 44607 (54.56) |
| Ex-smoker | 11913 (29.14) | 18318 (22.41) |
| Missing | 590 (1.44) | 5224 (6.39) |
| Ethnicity (%) |  |  |
| White | 15885 (38.86) | 33577 (41.07) |
| Mixed Race | 343 (0.84) | 529 (0.65) |
| Other | 147 (0.36) | 213 (0.26) |
| Black | 940 (2.30) | 860 (1.05) |
| South Asian | 1504 (3.68) | 1035 (1.27) |
| Missing | 22060 (53.96) | 45543 (55.71) |

**Supplementary Table S3: Crude and adjusted HRs for the risk of RA in subgroup of patients stratified by sex**.

|  | **Male cohort** | | **Female cohort** | |
| --- | --- | --- | --- | --- |
|  | **Exposed** | **Unexposed** | **Exposed** | **Unexposed** |
| Population | 125,558 | 251,116 | 98,993 | 197,985 |
| Outcome events, n (%) | 393 (0.31) | 896 (0.36) | 578 (0.58) | 1,221 (0.62) |
| Person-years | 673,098 | 1,090,674 | 526,943 | 908,627 |
| Crude Incidence Rate/1000 person years | 0.58 | 0.82 | 1.10 | 1.34 |
| Follow-up years, Median (IQR) | 4.54 (2.03-7.98) | 3.33 (1.38-6.43) | 4.47 (1.99-7.95) | 3.58 (1.51-6.84) |
| Unadjusted Hazard ratio (95% CI) | 0.72 (0.64-0.81) | | 0.83 (0.75-0.92) | |
| p-value | p<0.01 | | p<0.01 | |
| Adjusted Hazard ratio (95% CI) | 0.69 (0.61-0.79) | | 0.76 (0.68-0.85) | |
| p-value | p<0.01 | | p<0.01 | |

Model was adjusted for sex, age categories, BMI categories, Townsend deprivation quintiles, smoking status, and ethnicity. HR: hazard ratio; IQR: interquartile range; CI: confidence interval

**Supplementary Table S4: Crude and adjusted HRs for the risk of RA in patients with no history of statins use**

|  | **Exposed** | **Unexposed** |
| --- | --- | --- |
| Population | 40,879 | 81,757 |
| Outcome events, n (%) | 107 (0.26) | 232 (0.28) |
| Person-years | 145,387 | 310,679 |
| Crude Incidence Rate/1000 person years | 0.74 | 0.75 |
| Follow-up years, Median (IQR) | 2.57 (1.06-5.12) | 2.76 (1.15-5.42) |
| Unadjusted Hazard ratio (95% CI) | 0.99 (0.79-1.24) | |
| p-value | p=0.91 | |
| Adjusted Hazard ratio (95% CI) | 0.89 (0.69-1.14) | |
| p-value | p=0.34 | |

Model was adjusted for sex, age categories, BMI categories, Townsend deprivation quintiles, smoking status, and ethnicity. HR: hazard ratio; IQR: interquartile range; CI: confidence interval

**Supplementary Table S5: Risk of incident RA in diabetic patients using time-dependent analysis**

| **Variable** | **Hazard ratio** | **95% Confidence interval** | |
| --- | --- | --- | --- |
| Female | 1.70 | 1.48 | 1.96 |
| Age | 1.01 | 1.00 | 1.02 |
| BMI | 1.01 | 1.00 | 1.02 |
| HbA1C | 1.00 | 0.99 | 1.00 |
| EGFR | 1.00 | 1.00 | 1.00 |
| Systolic blood pressure | 1.00 | 0.99 | 1.00 |
| Deprivation *(ref: Townsend 1)* |  |  |  |
| Townsend 2 | 0.98 | 0.78 | 1.23 |
| Townsend 3 | 0.97 | 0.77 | 1.21 |
| Townsend 4 | 0.94 | 0.75 | 1.17 |
| Townsend 5 (most deprived) | 1.10 | 0.87 | 1.38 |
| Townsend 6 (Missing) | 0.94 | 0.74 | 1.19 |
| Smoking (*ref: Non-smoker*) |  |  |  |
| Smoker | 1.48 | 1.23 | 1.78 |
| Ex-smoker | 1.37 | 1.17 | 1.59 |
| Missing | 1.03 | 0.62 | 1.70 |
| Ethnicity *(ref: White)* |  |  |  |
| Mixed race | 0.43 | 0.11 | 1.74 |
| Other | 2.35 | 0.88 | 6.29 |
| Black | 0.80 | 0.41 | 1.55 |
| South Asian | 1.43 | 1.00 | 2.05 |
| Missing | 0.95 | 0.83 | 1.09 |
| Peripheral neuropathy | 0.91 | 0.67 | 1.25 |
| Retinopathy | 1.17 | 0.76 | 1.79 |
| Diabetic foot ulcer | 1.06 | 0.68 | 1.66 |
| Hypothyroidism | 1.35 | 1.10 | 1.66 |
| Cardiovascular disease | 0.86 | 0.72 | 1.00 |
| Metformin *(ref: Other oral drugs only)* |  |  |  |
| Metformin* | 1.00 | 0.82 | 1.22 |
| No oral drug | 0.97 | 0.73 | 1.29 |
| Statins | 0.76 | 0.66 | 0.88 |

* Metformin with or without other oral drugs, BMI: Body mass index, HbA1C: Haemoglobin A1C, EGFR: Estimated glomerular filtration rate

**Supplementary Table S6: Risk of incident RA in diabetic patients using time-dependent analysis and cumulative duration for statins**

| **Variable** | **Hazard ratio** | **95% Confidence interval** | |
| --- | --- | --- | --- |
| Female | 1.71 | 1.48 | 1.97 |
| Age | 1.01 | 1.00 | 1.02 |
| BMI | 1.01 | 1.00 | 1.02 |
| HbA1C | 1.00 | 0.99 | 1.00 |
| EGFR | 1.00 | 1.00 | 1.00 |
| Systolic blood pressure | 1.00 | 0.99 | 1.00 |
| Deprivation *(ref: Townsend 1)* |  |  |  |
| Townsend 2 | 0.98 | 0.78 | 1.22 |
| Townsend 3 | 0.96 | 0.77 | 1.20 |
| Townsend 4 | 0.93 | 0.75 | 1.17 |
| Townsend 5 (most deprived) | 1.09 | 0.87 | 1.38 |
| Townsend 6 (Missing) | 0.94 | 0.74 | 1.19 |
| Smoking (*ref: Non-smoker*) |  |  |  |
| Smoker | 1.47 | 1.22 | 1.77 |
| Ex-smoker | 1.36 | 1.16 | 1.59 |
| Missing | 1.03 | 0.62 | 1.71 |
| Ethnicity *(ref: White)* |  |  |  |
| Mixed race | 0.43 | 0.11 | 1.74 |
| Other | 2.36 | 0.88 | 6.33 |
| Black | 0.81 | 0.42 | 1.57 |
| South Asian | 1.44 | 1.01 | 2.05 |
| Missing | 0.96 | 0.83 | 1.09 |
| Peripheral neuropathy | 0.91 | 0.67 | 1.25 |
| Retinopathy | 1.17 | 0.77 | 1.79 |
| Diabetic foot ulcer | 1.07 | 0.68 | 1.67 |
| Hypothyroidism | 1.35 | 1.10 | 1.66 |
| Cardiovascular disease | 0.84 | 0.70 | 1.01 |
| Metformin *(ref: Other oral drugs only)* |  |  |  |
| Metformin* | 0.98 | 0.80 | 1.19 |
| No oral drug | 1.02 | 0.77 | 1.35 |
| Statins duration *(ref: No statin use)* |  |  |  |
| < 1.5 years | 0.96 | 0.79 | 1.17 |
| 1.5 - 3 years | 1.02 | 0.80 | 1.29 |
| 3 - 4.5 years | 0.77 | 0.57 | 1.05 |
| > 4.5 years | 0.75 | 0.57 | 0.98 |

*Metformin with or without other oral drugs, BMI: Body mass index, HbA1C: Haemoglobin A1C, EGFR: Estimated glomerular filtration rate

**Supplementary Table S7: Crude and adjusted HRs for the association between the use of DPP4 inhibitors and the risk of RA**

| **Variable** | **Hazard ratio** | **95% Confidence interval** | |
| --- | --- | --- | --- |
| Female | 1.62 | 1.36 | 1.93 |
| Age | 1.01 | 1.00 | 1.02 |
| BMI | 1.01 | 1.00 | 1.02 |
| HbA1C | 1.00 | 0.99 | 1.00 |
| EGFR | 1.00 | 0.99 | 1.00 |
| Systolic blood pressure | 1.00 | 0.99 | 1.00 |
| Deprivation *(ref: Townsend 1)* |  |  |  |
| Townsend 2 | 0.86 | 0.64 | 1.14 |
| Townsend 3 | 0.98 | 0.75 | 1.28 |
| Townsend 4 | 0.86 | 0.65 | 1.14 |
| Townsend 5 (most deprived) | 1.08 | 0.82 | 1.44 |
| Townsend 6 (Missing) | 0.94 | 0.71 | 1.23 |
| Smoking (*ref: Non-smoker*) |  |  |  |
| Smoker | 1.44 | 1.15 | 1.81 |
| Ex-smoker | 1.21 | 1.00 | 1.46 |
| Missing | 1.92 | 0.71 | 5.18 |
| Ethnicity *(ref: White)* |  |  |  |
| Mixed race | 0.56 | 0.14 | 2.27 |
| Other | 2.60 | 0.83 | 8.12 |
| Black | 0.65 | 0.27 | 1.57 |
| South Asian | 1.34 | 0.87 | 2.08 |
| Missing | 1.01 | 0.85 | 1.19 |
| Peripheral neuropathy | 0.97 | 0.69 | 1.36 |
| Retinopathy | 1.36 | 0.86 | 2.16 |
| Diabetic foot ulcer | 0.91 | 0.51 | 1.61 |
| Hypothyroidism | 1.45 | 1.14 | 1.84 |
| Cardiovascular disease | 0.72 | 0.57 | 0.90 |
| DPP4i *(ref: Other oral drugs only)* |  |  |  |
| DPP4i* | 0.94 | 0.71 | 1.24 |
| No oral drug | 1.01 | 0.77 | 1.33 |
| Statins | 0.79 | 0.66 | 0.94 |

*DPP4i with or without other oral drugs, DPP4i: Dipeptidyl peptidase-4 inhibitors, BMI: Body mass index, HbA1C: Haemoglobin A1C, EGFR: Estimated glomerular filtration rate

**STROBE Statement: Checklist of items that should be included in reports of cohort studies**

|  | **Item No** | **Recommendation** | **Reporting Location** |  |
| --- | --- | --- | --- | --- |
| **Title and abstract** | 1 | (*a*) Indicate the study’s design with a commonly used term in the title or the abstract | Abstract |  |
|  |  | (*b*) Provide in the abstract an informative and balanced summary of what was done and what was found | Abstract |  |
| **Introduction** | | |  |  |
| Background/rationale | 2 | Explain the scientific background and rationale for the investigation being reported | Introduction |  |
| Objectives | 3 | State specific objectives, including any prespecified hypotheses | Introduction |  |
| **Methods** | | |  |  |
| Study design | 4 | Present key elements of study design early in the paper | Methods |  |
| Setting | 5 | Describe the setting, locations, and relevant dates, including periods of recruitment, exposure, follow-up, and data collection | Methods |  |
| Participants | 6 | (*a*) Give the eligibility criteria, and the sources and methods of selection of participants. Describe methods of follow-up | Methods |  |
|  |  | (*b*) For matched studies, give matching criteria and number of exposed and unexposed | Methods  Results: Table 1 |  |
| Variables | 7 | Clearly define all outcomes, exposures, predictors, potential confounders, and effect modifiers. Give diagnostic criteria, if applicable | Methods |  |
| Data sources/ measurement | 8 | For each variable of interest, give sources of data and details of methods of assessment (measurement). Describe comparability of assessment methods if there is more than one group | Methods |  |
| Bias | 9 | Describe any efforts to address potential sources of bias | Methods/discussion |  |
| Study size | 10 | Explain how the study size was arrived at | Methods |  |
| Quantitative variables | 11 | Explain how quantitative variables were handled in the analyses. If applicable, describe which groupings were chosen and why | Methods |  |
| Statistical methods | 12 | (*a*) Describe all statistical methods, including those used to control for confounding | Methods |  |
|  |  | (*b*) Describe any methods used to examine subgroups and interactions | Methods |  |
|  |  | (*c*) Explain how missing data were addressed | Methods |  |
|  |  | (*d*) If applicable, explain how loss to follow-up was addressed | Methods |  |
|  |  | (*e*) Describe any sensitivity analyses | Methods and results |  |
| **Results** | | |  |  |
| Participants | 13 | (a) Report numbers of individuals at each stage of study—eg numbers potentially eligible, examined for eligibility, confirmed eligible, included in the study, completing follow-up, and analysed | Methods and results |  |
|  |  | (b) Give reasons for non-participation at each stage | N/A |  |
|  |  | (c) Consider use of a flow diagram |  |  |
| Descriptive data | 14 | (a) Give characteristics of study participants (eg demographic, clinical, social) and information on exposures and potential confounders | Results |  |
|  |  | (b) Indicate number of participants with missing data for each variable of interest | Table 1 |  |
|  |  | (c) Summarise follow-up time (eg, average and total amount) | Table 2 and supplementary tables |  |
| Outcome data | 15 | Report numbers of outcome events or summary measures over time | Table 2 and supplementary tables |  |
| Main results | 16 | (*a*) Give unadjusted estimates and, if applicable, confounder-adjusted estimates and their precision (eg, 95% confidence interval). Make clear which confounders were adjusted for and why they were included | Table 2 and supplementary tables |  |
|  |  | (*b*) Report category boundaries when continuous variables were categorized | Table 1 |  |
|  |  | (*c*) If relevant, consider translating estimates of relative risk into absolute risk for a meaningful time period | N/A |  |
| Other analyses | 17 | Report other analyses done—eg analyses of subgroups and interactions, and sensitivity analyses | Results |  |
| **Discussion** | | |  |  |
| Key results | 18 | Summarise key results with reference to study objectives | Discussion |  |
| Limitations | 19 | Discuss limitations of the study, taking into account sources of potential bias or imprecision. Discuss both direction and magnitude of any potential bias | Discussion |  |
| Interpretation | 20 | Give a cautious overall interpretation of results considering objectives, limitations, multiplicity of analyses, results from similar studies, and other relevant evidence | Discussion |  |
| Generalisability | 21 | Discuss the generalisability (external validity) of the study results | Discussion |  |
| **Other information** | | |  |  |
| Funding | 22 | Give the source of funding and the role of the funders for the present study and, if applicable, for the original study on which the present article is based | Title/acknowledgement page |  |
